# SARS-CoV-2 quasi-species analysis from patients with persistent nasopharyngeal shedding

**DOI:** 10.1038/s41598-022-22060-z

**Published:** 2022-11-04

**Authors:** Pierre Dudouet, Philippe Colson, Sarah Aherfi, Anthony Levasseur, Mamadou Beye, Jeremy Delerce, Emilie Burel, Philippe Lavrard, Wahiba Bader, Jean-Christophe Lagier, Pierre-Edouard Fournier, Bernard La Scola, Didier Raoult

**Affiliations:** 1 IHU Méditerranée Infection, 19-21 Boulevard Jean Moulin, 13005 Marseille, France; 2 Microbes Evolution Phylogeny and Infections (MEPHI), Institut de Recherche Pour Le Développement (IRD), Assistance Publique-Hôpitaux de Marseille (AP-HM), MEPHI, Aix-Marseille Univ., 27 Boulevard Jean Moulin, 13005 Marseille, France; 3 Vecteurs-Infections Tropicales et Méditerranéennes (VITROME), Aix-Marseille Univ, Marseille, France

**Keywords:** Clinical genetics, Evolutionary biology, Genomics, Haplotypes, Sequencing, SARS-CoV-2

## Abstract

At the time of a new and unprecedented viral pandemic, many questions are being asked about the genomic evolution of SARS-CoV-2 and the emergence of different variants, leading to therapeutic and immune evasion and survival of this genetically highly labile RNA virus. The nasopharyngeal persistence of infectious virus beyond 17 days proves its constant interaction with the human immune system and increases the intra-individual mutational possibilities. We performed a prospective high-throughput sequencing study (ARTIC Nanopore) of SARS-CoV-2 from so-called "persistent" patients, comparing them with a non-persistent population, and analyzing the quasi-species present in a single sample at time t. Global intra-individual variability in persistent patients was found to be higher than in controls (mean 5.3%, Standard deviation 0.9 versus 4.6% SD 0.3, respectively, *p* < 0.001). In the detailed analysis, we found a greater difference between persistent and non-persistent patients with non-severe COVID 19, and between the two groups infected with clade 20A. Furthermore, we found minority N501Y and P681H mutation clouds in all patients, with no significant differences found both groups. The question of the SARS-CoV-2 viral variants’ genesis remains to be further investigated, with the need to prevent new viral propagations and their consequences, and quasi-species analysis could be an important key to watch out.

## Introduction

Acute infectious respiratory diseases is one of the main causes of morbidity and mortality worldwide, and viral infections of lower respiratory tract account for a large proportion^1^. Among them, coronaviruses are the largest group of non-segmented, single-stranded, positive-sense ribonucleic acid viruses (+ ssRNA)^2^. They belong to the order Nidovirales, family Coronaviridae, subfamily Coronavirinae, and cause zoonotic infections in many vertebrates^3^. In December 2019, a new coronavirus, severe acute respiratory syndrome-Coronavirus-2 (SARS-CoV-2), was reported for the first time in the city of Wuhan, Hubei Province, China, causing a rapidly pandemic severe infection in humans (COVID 19). SARS-CoV-2 was sequenced as an enveloped ssRNA virus with a complete genomic sequence containing 29,903 nucleotides and encoding 7986 amino acids^4^. Phylogenetic analysis of coronavirus genomes has revealed that SARS-CoV-2 belongs to subgenus *Sarbecovirus* in genus *Betacoronavirus*, with high similarity (96%) to bat *betacoronavirus* RaTG13, suggesting its potential zoonotic origin^5^.

Like other RNA viruses, beta-coronaviruses can have complex and dynamic cycles of genomic variation within a population or within a single host, and thus exhibit significant polymorphism ^6^. The rate of evolution of SARS-CoV-2 is considered moderate, estimated at 1.19–1.31 × 10^3^ substitutions per site per year^6^, which tends to increase today to around 2.68–3.86 × 10^3^ per site per year, mainly due to the low fidelity of its RdRp, which could evolve with time^7^. Thus, new mutants, clones, and then viral variants born from each infected host, having different infectivity and contagiousness and playing in an incredible way on the evolution of the different epidemic currents of COVID-19^8^. As an example, a link between increased mutations and treatment has recently been demonstrated, as well as the selection pressure of the host immune system, associated with more mutations in spike domain^9^. It may suggest, however, that the origin of these new inter-individual viral entities called "variants" is more subtle as several teams have, in an analogous way to HIV or other RNA viruses, studied the possibility of the existence of significant intra-individual variability leading to this genetic polymorphism^10^. Advent of next generation sequencing (NGS) techniques has allowed identification of theses intra-individual viral subpopulations, called quasispecies, in patients infected with SARS-CoV^11^ and MERS-CoV^12^, suggesting their existence for SARS-CoV-2 yet^7^ with an estimated average genetic distance of ~ 8.36 × 10^–4^. The presence of these SARS-CoV-2 viral quasi-species was thus observed in various types of biological samples, particularly nasopharyngeal, with minority variants distributed evenly along the genome ranging in frequency from 1 to ~ 30%^13^. Appearance of viral variants has now been strongly suggested to be an indirect consequence of finest intra-individual genetic evolutions, and hence, fair questions are rising about accountability in this mechanism^14^. Information on literature is missing, first, regarding the effect of anti-SARS-CoV-2 treatments and vaccines on mutability, but also regarding clinical risk factors to become a “variant maker”, while prevention of escaping mutations in the framework of a genomic watch has become indispensable.

Among others, the question of persistence of SARS-CoV-2 viral excretion is not well defined yet and could potentially accelerate genomic viral evolutions^15^. Two meta-analyses, including 79 and 28 studies, converged to indicate a naso-pharyngeal viral shedding duration of 17 days (mean) and 18.4 days (median), respectively^16,17^. Viral persistence defined as longer viral shedding (> 17 days) has concerned about 30% of patients during the initial outbreak with the WU strain, mainly immunocompromised, with comorbidities, or a severe clinical stage^18^, but also recently with emerging VOCs such as Omicron 21 K (https://wwwnc.cdc.gov/eid/article/28/5/22-0197_article).

Significant differences in cytokine profiles and immune transcriptomes between persistent and non-persistent patient populations also exist, associated with a longer host–pathogen interaction, and consequently a higher mutational risk^19^. It is thus legitimate to assess the role of these persistent patients in the evolution of SARS-CoV-2, both by the presence of a longer transmission risk, and by that of pre-existing mutation fixation in the viral sub-population.

In this context, we conducted a prospective study on 160 nasopharyngeal PCR-positive SARS-CoV-2 samples to assess the possible differences in the intra-individual genetic variability between persistent and non-persistent patients. Primary endpoint being the mean intra-individual genomic variability compared between the so-called "persistent" and "non-persistent" patient populations. As secondary endpoint, we also analyzed intra-host variation in spike gene, we analyzed in detail the most variable genomic positions and patients and investigated whether mutations of interest currently present were already present in viral subpopulations before they spread.

## Results

### Characteristics of patients

**Figure 1:**
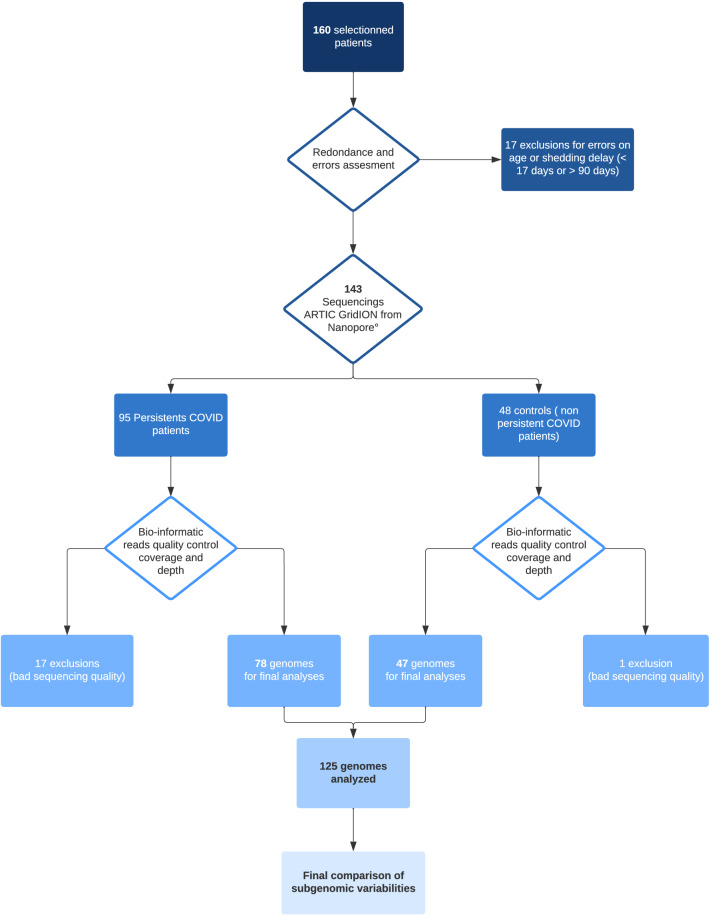
Flowchart.

A total of 160 samples were collected, divided into 105 persistent and 55 non-persistent patients (control group). After clinical data analysis from persistent group, 17 patients were excluded, 14 for errors on persistence viral shedding (below 17 days), and 3 patients for being below 18 years old of age (Fig. 1). After quality sequence analyzing, bad sequencings were found in 17 patients from persistent group and 1 from control group. Among persistent patients, mean age was 67 years old (SD 17.8) and there were 63% of men. Mean shedding delay was measured at 26 (+ /− 6) days. Immunodepression background was divided into five sections: 0: none (46%); 1: diabete mellitus (29%); 2: hemopathies (5%); 3: solid organ graft with immunosuppressors (6.5%); 4: active solid cancer with chemotherapy or immunotherapy (6.5%) and 5: autoimmune disease with immunotherapy (3.9%). We do not have background data for 2.6% of patients (n = 2). Main patients received specific SARS-CoV-2 treatment (75%) among them azithromycin, hydroxychloroquine, dexamethasone, ivermectin, alone or in association (Table 1). Antibiotic therapy against bacterial infections was not assessed. Severity of the disease was divided into 4 items according to the maximum stage reached by patient: 1: mild stage (ambulatory); 2: moderate stage (hospitalization); 3: severe stage (intensive care unit); 4: death. Thus, we had 27.8% of patients on stage 1 and 3, 25% on stage 2 and 2.8% on stage 4. Missing data concerned 6.5% of patients. No differences were found between persistent group and control group in propension matched multivariate analyses, Except for age, disease stage 3 and ICU admission (Table 1).

**Table 1:** Characteristics of patients.

Characteristics	Persistent (n = 91); n (%)	Non-persistent (n = 47); n (%)	*p*
**Sex ratio**			
*Men*	59 (63)	33 (67)	*0.092*
**Age (mean, SD)**	67 (17)	49 (19)	***0.004***
**Immunodepression background**			
*0: None*			*0.067*
*1: Diabetes mellitus*	35 (46)	28 (48)	
*2: Malign hemopathies*	22 (29)	4 (7)	
*3: Solid organ graft*	4 (5)	0 (0)	
*4: Active organ solid malignancy with CT or IT*	5 (6)	2 (3)	
*5: auto-immune disease treated by IT or IST*	5 (6)	3 (5)	
*Missing data*	3 (4)	3 (5)	
	2 (2)	20 (30)	
**COVID 19 treatments**			*0.151*
*AZ alone*	5 (5)	4 (6)	
*AZ* + *DXM*	5 (5)	1 (2)	
*AZ* + *IVE*	1 (1)	0 (0)	
*HCQ* + *AZ*	18 (9)	18 (30)	
*HCQ* + *AZ* + *DXM*	7 (8)	0 (0)	
*DXM alone*	16 (17)	7 (12)	
*Anti C5a*	1 (1)	0 (0)	
*Missing data*	7 (8)	8 (13)	
*No specific treatment*	28 (30)	22 (36)	
**Severity stage of disease**			
*1: mild*	21 (22)	21 (35)	*0.092*
*2: moderate*	19 (21)	11 (18)	*0.741*
*3: severe*	21 (22)	3 (5)	***0.022***
*4: death*	9 (10)	1 (2)	*0.065*
*Missing data*	27 (29)	22 (36)	
**ICU admission**			
*Yes*	25 (26)	4 (7)	***0.018***
*No*	47 (50)	34 (56)	*0.714*
*Missing data*	21 (23)	20 (30)	

*IT: immunotherapy; IST: immunosuppressive therapy; AZ: azithromycin; DXM: dexamethasone; HCQ: hydroxychloroquine; IVE: ivermectin; ICU: intensive care unit.*

Significant values are in bold and italics.

### Characteristics of sequencings

After clinical data analysis, we have sequenced 144 SARS-CoV-2 samples from nasopharyngeal swab. Mean genome coverage was measured at 90.7% (+ /− 12.5), median 99.7%, and mean depth per position at 1.738 reads (+ /− 1.065). SARS-CoV-2/human reads ratio was as follows: median 0.89, mean 0.79 (+ /− 0.25). Whole genome quality was also assessed on Nextstrain and Auspice (Supplementary Fig. 2). 12 sequencings were excluded for too low coverage, 11 in persistent group, 1 in control group. Details on sequencing-including additional mutations-are notified on Supplementary Table 2. According to Nextstrain analysis, we have obtained a clade distribution consisting of 47% and 25% of 20A, 15% and 6% of 20E, 19% and 32% of 20I/Alpha variant, 12% and 32% of 20 J/Beta variant, 7% and 6% of other clades, in persistent and non-persistent groups, respectively (Supplementary Fig. 2).

In the aim to assess risk of bias from ARTIC amplification, we analyzed read distribution and observe on linear regression a negative correlation between Ct value and number of reads per position (R = 0.44, *p* < 0.001), between mean variability per sample and number of reads per position per sample, and a positive correlation between Ct and mean variability per sample (R = 0.29, *p* < 0.001). Thus, position with a high number of reads does not wrongly reflect high variability. (Supplementary Fig. 3).

### Comparison between variabilities from persistent and non-persistent patients in whole genomes and in Spike domain

**Figure 2:**
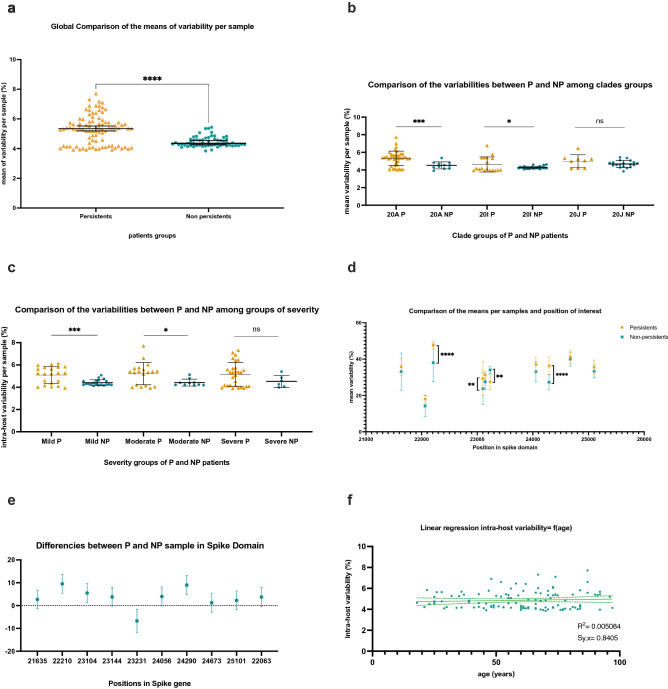
Comparison of intra-host variability among several criteria between samples from persistent (yellow) and non-persistent (blue) patients. (**a**) global comparison of intra-host variability per sample. Welch’s t comparison test. (**b**) differences between clades. Kolmogorov–smirnov comparison test. (**c**) differences between severity of COVID-19. Unpaired t test used. (**d**) Hotspot mutations in spike domain. Mixed effect analysis, Šídák's multiple comparisons test. (**e**)Variance’s comparison in spike domain. Mixed effect analysis, Šídák's multiple comparisons test. (**f**) linear regression between intra-host variability and age. Simple linear regression test.

**Figure 3:**
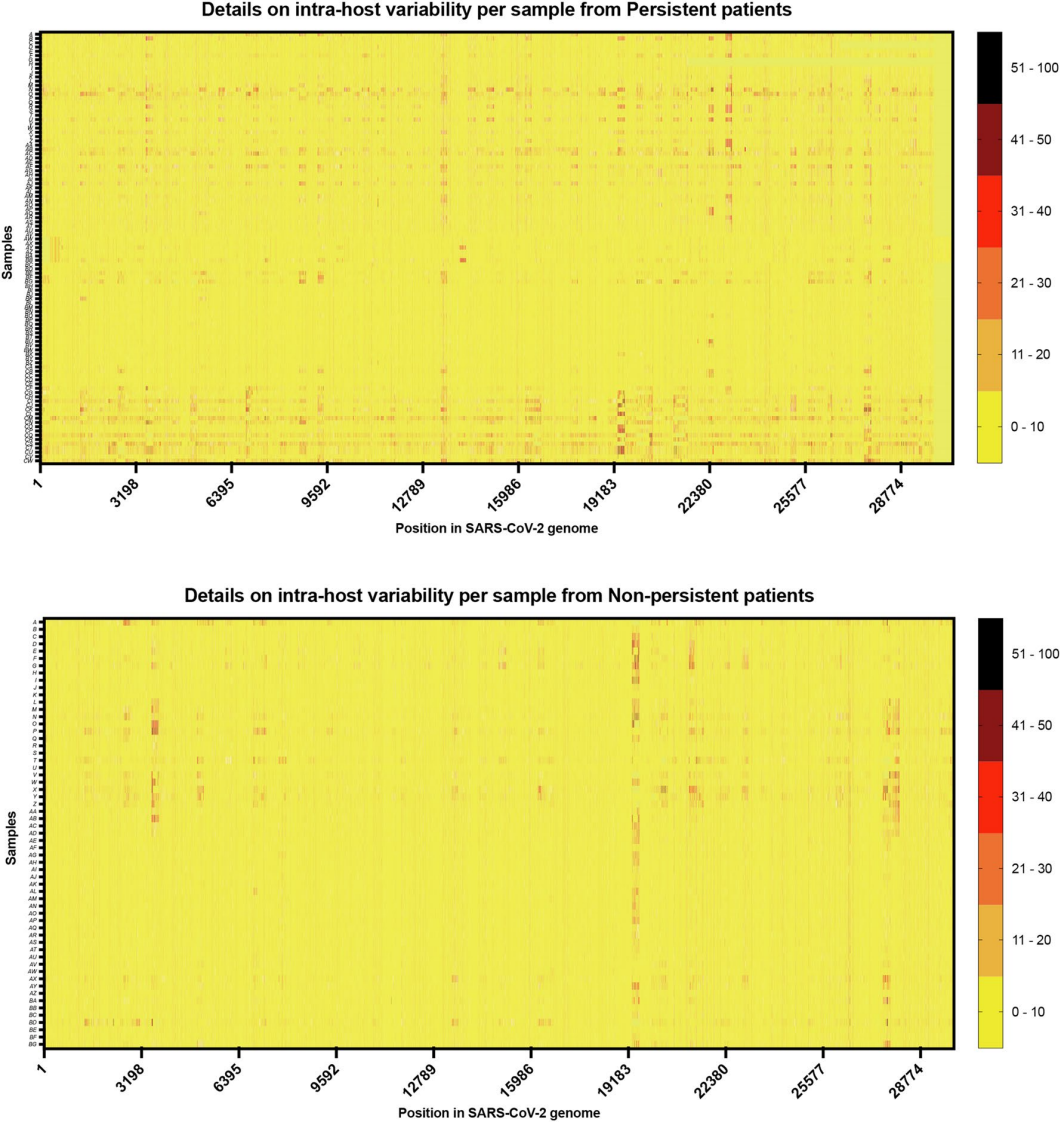
Details on variability for each sequenced samples (Y rows) by position in SARS-CoV-2 genome (X columns). Scale of variability is described at the right.

In global analysis (Fig. 2a), the mean intra-host variability for all samples and in the whole genomes was 5.4% (SD 0.9%) in persistent group versus 4.6% (SD 0.3%) in control group, with significant difference of the means and variances found on unpaired t-test analysis with Welch correction (−0.67 ± 0.12; *p* < 0,001). Within clades groups analysis (Fig. 2b), the intra-host variability was significantly different and higher between persistent and non-persistent samples from clades 20A and 20I (*p* = 0.009 and *p* = 0.019 respectively), but not from clade 20 J (*p* = 0.15). Within severity groups analysis (Fig. 2c), no differences on means were found between persistent and non-persistent patients suffering from severe-clinical stage 3 and 4-COVID 19 (5146 *vs* 4522, *p* = 0.17), whereas significant differences occurred between persistent and non-persistent patients from mild and moderate clinical group (5019 vs 4143, *p* = 0.0005 and 5222 *vs* 4414 *p* = 0.019, respectively). In spike gene (positions 21,563–25,384), we found ten super-variable positions (21,635; 22,063; 22,210; 23,104; 23,144; 23,231; 24,056; 24,290; 24,673 and 25,101). Four showed significant mean differences: 22,210; 23,104 and 24,056 harbored increasing variability in persistent sample (differences between means: + 9.5 *p* < 0.001; + 5.5 *p* = 0.002; + 8.9, *p* < 0.001 respectively), while variability was more important in non-persistent samples on position 23,231 (difference between means −6.7, *p* = 0.0017) (Fig. 2d,e). We did not find any correlation between age and intra-host variability on simple linear regression test, with R2 equal to 0.009840 and Sy.x equal to 0.83 (Fig. 2f). Global representation of variability per sample and for the whole genome is given in Fig. 3.

### Description of hot-spot positions

**Figure 4:**
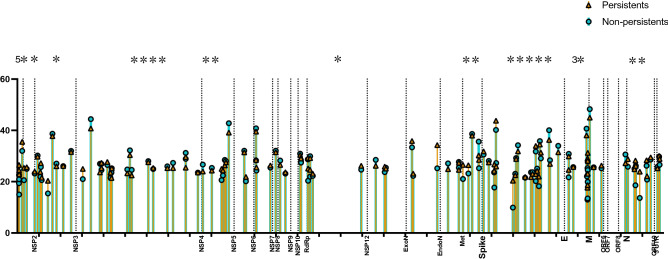
Hotspot position chosen in sample with median of variability for a given position was higher than 25%. One bare represents the mean of variability for one position, blue bars and circle for non-persistent samples, and yellow bars for persistent samples.

A total of 123 hot-spot positions were found, 5 positions located in 5’UTR gene, 3 in NSP1, 9 in NSP2, 19 in NSP3, 7 in NSP4, 2 in NSP5 and NSP8, 4 in NSP6 and NSP10, 6 in RdRp, 3 in Helicase, Endonuclease, Exonuclease and Methylase domains, 22 in spike gene, 11 in gene “E”, 3 in genes M and ORF8, 8 in gene “M” and 3 in 3’UTR domain (Fig. 4 and Table 2). Comparing P and NP samples, only 25 positions showed significant differences, with more differences in persistent group, 5 in 5’UTR, 1 in NSP1 and NSP2, 4 in NSP3 gene, 2 in NSP4, 1 in RdRp, 2 in Methylase gene, 5 in spike domain, 3 in gene “E” and 2 in gene “N” (Fig. 4 (stars); Table 2). Significant differences showing higher intra-host variations in favor of non-persistent samples have been found in positions 3833; 7814; 21,409; 24,673; 26,562 and 28,215 positions (6 out of 25).

**Table 2:** Uncorrected Fisher's LSD test comparing differences between persistent and non-persistent (on way) in positions showing a median of variability higher than 25%. Spike domain in bold character. CI: confidence interval; LSD: least significant difference; Diff.: differences.

Position in SARS-CoV-2 genome	Predicted mean diff	95.00% CI of diff	Below threshold?	Summary	Individual *P* Value
77	3.892	0.9903 to 6.793	Yes	**	*0.0086*
78	3.578	0.6766 to 6.479	Yes	*	*0.0157*
79	3.888	0.9865 to 6.789	Yes	**	*0.0086*
84	7.875	4.973 to 10.78	Yes	****	< *0.0001*
227	3.568	0.6669 to 6.470	Yes	*	*0.0159*
307	4.855	1.954 to 7.757	Yes	**	*0.0010*
435	0.6604	− 2.212 to 3.533	No	ns	*0.6522*
803	0.7572	− 2.115 to 3.629	No	ns	*0.6054*
942	− 0.3859	− 3.258 to 2.486	No	ns	*0.7923*
1067	1.868	− 1.004 to 4.741	No	ns	*0.2023*
1091	1.513	− 1.359 to 4.385	No	ns	*0.3018*
1131	0.9277	− 1.945 to 3.800	No	ns	*0.5267*
1420	4*.*918	2.046 to 7.791	Yes	***	*0.0008*
1629	− 0.9281	− 3.800 to 1.944	No	ns	*0.5265*
1814	− 1.049	− 3.921 to 1.823	No	ns	*0.4741*
2130	0.07450	− 2.798 to 2.947	No	ns	*0.9595*
2494	− 0.4290	− 3.301 to 2.443	No	ns	*0.7697*
3037	4.013	1.141 to 6.886	Yes	**	*0.0062*
3413	− 3.637	− 6.509 to − 0.7649	Yes	*	*0.0131*
3833	− 3.312	− 6.184 to − 0.4392	Yes	*	*0.0238*
3902	0.7680	− 2.104 to 3.640	No	ns	*0.6002*
3903	0.4281	− 2.444 to 3.300	No	ns	*0.7702*
4175	1.207	− 1.665 to 4.079	No	ns	*0.4102*
4322	2.025	− 0.8476 to 4.897	No	ns	*0.1671*
4370	− 2.125	− 4.997 to 0.7477	No	ns	*0.1471*
4383	− 0.02589	− 2.898 to 2.846	No	ns	*0.9859*
5100	− 1.508	− 4.385 to 1.369	No	ns	*0.3043*
5225	− 1.835	− 4.712 to 1.042	No	ns	*0.2113*
5305	− 2.140	− 5.012 to 0.7327	No	ns	*0.1443*
6078	− 0.4271	− 3.299 to 2.445	No	ns	*0.7707*
6306	0.4119	− 2.460 to 3.284	No	ns	*0.7786*
6962	− 0.7172	− 3.604 to 2.169	No	ns	*0.6262*
7225	− 1.991	− 4.864 to 0.8809	No	ns	*0.1742*
7814	− 3.393	− 6.265 to − 0.5204	Yes	*	*0.0206*
7815	− 1.610	− 4.482 to 1.263	No	ns	*0.2721*
8377	0.07948	− 2.793 to 2.952	No	ns	*0.9567*
8607	− 2.670	− 5.542 to 0.2024	No	ns	*0.0685*
9027	− 1.021	− 3.893 to 1.852	No	ns	*0.4861*
9475	4.372	1.499 to 7.244	Yes	**	*0.0029*
9539	1.957	− 0.9150 to 4.830	No	ns	*0.1817*
9628	− 2.102	− 4.975 to 0.7700	No	ns	*0.1514*
9681	− 0.4431	− 3.315 to 2.429	No	ns	*0.7624*
9812	− 3.660	− 6.533 to − 0.7881	Yes	*	*0.0125*
10,528	− 0.6040	− 3.476 to 2.268	No	ns	*0.6802*
10,606	1.681	− 1.192 to 4.553	No	ns	*0.2515*
11,075	− 1.257	− 4.129 to 1.616	No	ns	*0.3912*
11,076	0.1884	− 2.684 to 3.061	No	ns	*0.8977*
11,096	1.235	− 1.637 to 4.107	No	ns	*0.3993*
11,743	0.8390	− 2.033 to 3.711	No	ns	*0.5670*
11,991	− 0.5595	− 3.432 to 2.313	No	ns	*0.7026*
12,197	− 1.699	− 4.571 to 1.174	No	ns	*0.2464*
12,437	0.5408	− 2.331 to 3.413	No	ns	*0.7121*
13,124	− 0.5118	− 3.384 to 2.360	No	ns	*0.7269*
13,163	1.664	− 1.208 to 4.536	No	ns	*0.2562*
13,164	1.644	− 1.229 to 4.516	No	ns	*0.2620*
13,476	0.5222	− 2.350 to 3.394	No	ns	*0.7216*
13,492	4.814	1.941 to 7.686	Yes	**	*0.0010*
13,584	2.656	− 0.2166 to 5.528	No	ns	*0.0700*
13,587	− 0.7122	− 3.584 to 2.160	No	ns	*0.6270*
13,709	0.8719	− 2.000 to 3.744	No	ns	*0.5519*
15,955	1.539	− 1.333 to 4.411	No	ns	*0.2936*
16,631	− 2.319	− 5.200 to 0.5629	No	ns	*0.1148*
17,045	2.137	− 0.7350 to 5.010	No	ns	*0.1447*
17,100	− 0.2866	− 3.159 to 2.586	No	ns	*0.8449*
18,315	2.474	− 0.3982 to 5.346	No	ns	*0.0914*
18,369	0.8906	− 1.982 to 3.763	No	ns	*0.5434*
19,484	9.076	6.070 to 12.08	Yes	****	< *0.0001*
19,984	− 2.243	− 5.145 to 0.6590	No	ns	*0.1298*
20,487	− 0.3043	− 3.177 to 2.568	No	ns	*0.8355*
20,488	− 0.8657	− 3.738 to 2.007	No	ns	*0.5547*
20,679	5.523	2.651 to 8.396	Yes	***	*0.0002*
20,931	3.226	0.3535 to 6.098	Yes	*	*0.0277*
21,102	− 0.6568	− 3.529 to 2.215	No	ns	*0.6540*
21,409	− 5.408	− 8.294 to − 2.521	Yes	***	*0.0002*
21,422	3.274	0.3873 to 6.160	Yes	*	*0.0262*
**21,635**	**1.193**	** − 1.684 to 4.070**	**No**	**ns**	***0****.****4162***
**21,876**	** − 0.5915**	** − 3.473 to 2.290**	**No**	**ns**	***0****.****6874***
**22,121**	**0.6094**	** − 2.272 to 3.491**	**No**	**ns**	***0****.****6785***
**22,131**	**8.987**	**6.106 to 11.87**	**Yes**	****	** < *0.0001***
**22,210**	**3.617**	**0.7354 to 6.499**	**Yes**	*	***0****.****0139***
**22,219**	** − 1.356**	** − 4.238 to 1.525**	**No**	**ns**	***0****.****3563***
**22,992**	**10.50**	**7.623 to 13.39**	**Yes**	****	** < *0.0001***
**23,104**	** − 0.5266**	** − 3.408 to 2.355**	**No**	**ns**	***0****.****7202***
**23,144**	** − 0.3490**	** − 3.231 to 2.533**	**No**	**ns**	***0****.****8124***
**23,231**	** − 2.356**	** − 5.238 to 0.5257**	**No**	**ns**	***0****.****1091***
**23,561**	**0.2884**	** − 2.593 to 3.170**	**No**	**ns**	***0****.****8445***
**23,836**	** − 1.838**	** − 4.720 to 1.044**	**No**	**ns**	***0****.****2113***
**23,904**	** − 0.6141**	** − 3.496 to 2.267**	**No**	**ns**	***0****.****6761***
**24,056**	**2.194**	** − 0.6872 to 5.076**	**No**	**ns**	***0****.****1355***
**24,057**	**2.740**	** − 0.1415 to 5.622**	**No**	**ns**	***0****.****0624***
**24,120**	** − 0.8981**	** − 3.780 to 1.984**	**No**	**ns**	***0****.****5413***

Uncorrected Fisher's LSD	Predicted (LS) mean diff	95.00% CI of diff	Below threshold?	Summary	Individual *P* Value
**24,199**	**3.730**	**0.8489 to 6.612**	**Yes**	*	***0****.****0112***
**24,245**	** − 1.297**	** − 4.179 to 1.584**	**No**	**ns**	***0****.****3775***
**24,290**	**2.245**	** − 0.6370 to 5.126**	**No**	**ns**	***0****.****1268***
**24,673**	** − 3.784**	** − 6.666 to − 0.9027**	**Yes**	*	***0****.****0101***
**24,718**	** − 1.418**	** − 4.299 to 1.464**	**No**	**ns**	***0****.****3349***
**25,101**	** − 2.456**	** − 5.337 to 0.4261**	**No**	**ns**	***0****.****0949***
25,583	2**.**934	0.05215 to 5.815	Yes	*	*0.0460*
25,588	− 0**.**9908	− 3.872 to 1.891	No	ns	*0.5003*
25,798	0**.**09817	− 2.783 to 2.980	No	ns	*0.9468*
26,409	− 2**.**629	− 5.511 to 0.2525	No	ns	*0.0737*
26,431	0**.**9982	− 1.883 to 3.880	No	ns	*0.4971*
26,453	4**.**665	1.783 to 7.546	Yes	**	*0.0015*
26,455	3**.**789	0.9075 to 6.671	Yes	**	*0.0100*
26,461	5**.**185	2.303 to 8.066	Yes	***	*0.0004*
26,465	3**.**183	0.3018 to 6.065	Yes	*	*0.0304*
26,466	3**.**611	0.7298 to 6.493	Yes	*	*0.0140*
26,467	3*.*442	0.5600 to 6.323	Yes	*	*0.0192*
26,562	− 3*.*316	− 6.197 to − 0.4341	Yes	*	*0.0241*
26,746	0**.**08451	− 2.807 to 2.976	No	ns	*0.9543*
27,103	1*.*040	− 1.851 to 3.931	No	ns	*0.4808*
28,215	− 3**.**411	− 6.302 to − 0.5192	Yes	*	*0.0208*
28,331	2**.**988	0.09622 to 5.879	Yes	*	*0.0428*
28,637	− 0*.*1477	− 3.039 to 2.744	No	ns	*0.9203*
28,681	9**.**645	6.753 to 12.54	Yes	****	< *0.0001*
28,699	0.009643	− 2.882 to 2.901	No	ns	*0.9948*
28,881	10**.**29	7.402 to 13.18	Yes	****	< *0.0001*
29,196	2**.**127	− 0.7647 to 5.018	No	ns	*0.1494*
29,219	1**.**347	− 1.544 to 4.239	No	ns	*0.3610*
29,387	0**.**8119	− 2.089 to 3.713	No	ns	*0.5833*
29,701	− 0.5959	− 3.487 to 2.295	No	ns	*0.6863*
29,777	0**.**3283	− 2.563 to 3.220	No	ns	*0.8239*
29,801	2**.**255	− 0.6366 to 5.146	No	ns	*0.1264*

Significant values are in bold and italics.

### Presence of intra-host N501Y and P681H variants in 20A clade samples

**Figure 5:**
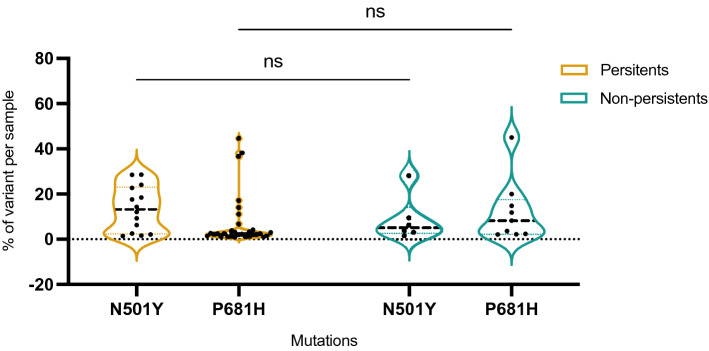
Mutant cloud (non on major quantity) found on specific 23,063 and 23,604 positions, corresponding to N501Y and P681H mutations assessing in patients infected from clades 20A only. Comparison between P and non-P for those position does not show significant differences.

We assessed only clade 20A, which do not contain any of N501Y neither P681H mutations, from our sample cohort to find those mutations in intra-host variants. There were 35 clade 20A within samples from P patients and 10 clade 20A within samples from NP patients. In P samples N501Y mutation was present in minor variant for 15 out of 35 P samples (43%), in a range from 1.6 to 28.6% of variants per sample (median: 15.9%). P681H mutation was, in turn, present in minor variant for 28 out of 35 P samples (80%), in a range from 1.1 to 44.6% of variants per sample (median: 2.5%). In the NP population, there were 10 samples from Clade 20A, and we found 6 N501Y variants (60%), with a median at 3.9%, and 8 P681H variants (80%) with a calculated median at 5.9% (Fig. 5). With ANOVA statistic settings, we could not find any significant differences between P and NP samples (*p* = 0.63 for N501Y mutation and *p* = 0.45 for P681H mutation).

## Discussion

Mutation’s origins in SARS-CoV-2 evolution are hard to assess, and especially to prevent, as shown *Wu *et al. Chinese’s team in a work where rising mutations and interacts with host immune system were represented in a one year retrospective eye^20^. Quasi-species, well studied in HIV advances, remains challenging current research on SARS-CoV-2 because of its propensity to see behind mutations, to see deeper in genomic flows, further than consensus sequence^14^. What is very interesting about what is described as a "cloud of viral mutants" is the way in which these populations are intrinsically selected. The pathogenesis was well described by *Domingo *et al. in 2019 in other RNA viruses, as an addition of micro-evolutionary events creating rich phenotypic intra-host reservoir, moving between dominance between variant clouds and interaction within host and intra-mutant spectra^21^. About SARS-CoV-2, studies on quasi-species are rare, but trend to put quasi-species as the number one suspect of mutational genesis^22^.

We here describe a large SARS-CoV-2 quasi-species study, in a relatively early population of viruses in the pandemic, notably before the appearance of the large monophyletic Variant of concerns (VOCs) delta and Omicron, and we suggest in our persistent population the higher ability to ad hidden nucleotide events in crucial positions. Persistent COVID-19, as we said above, is a rising entity suggesting high intra-host variability and concerning immune-injured population^19^. A recent study, *Perez-Lago *et al. have shown remarkable SARS-CoV-2 intra-host variability in three persistent shedding cases with time evolution^23^. They saw mutations rising from genomic weaknesses, especially in Spike and ORF1ab domains. This finding converges with our results since the most variable positions in our cohort and those that differed from NP were in the Spike and NSP3 domains. NSP3 gene, which code for Papain-like protease (PLpro), has been shown to have important function on host interactions, by ubiquitin-like action on inflammatory response and evasion from type 1 β-Interferon immune role^24^. Proofs are rising also concerning PLpro function in viral spreading control^25^. As persistence of viral shedding is linked with those host-pathogens interactions, we can extrapolate our results saying a higher intra-host variability might be due to those interactions, rather than the contrary.

In additional, intra-host variability was especially discovered, in our cohort, in persistent viral shedding patients. We particularly detected the same type of subvariant’s mutations (deletion, transversion, transition) in persistent and non-persistent samples, but in a higher percentage per position in persistent samples. Even if common quasi-species analyses are studied within a genomic evolutional timeline composed by several samples in the same patients, we have chosen a different way, shot gunning quasi-species at a t-time from on patient sample. Most of the subvariants cloud modifications found in persistent samples were deletions or synonymous mutations, as in several studies on quasi-species^26–29^, which could suggest natural correction and vanishing of those potential sources of mutation. But, it exists a potential silent role of synonymous mutations, as Khateeb et al. described significant reduction of infectivity and escape from BNT162b2 vaccine in minor part of pseudo viruses nasal population, but with a major synonymous mutation composition^30^.

In our spike gene analysis (positions 21,563–25,384), ten super-variable positions (21,635; 22,063; 22,210; 23,104; 23,144; 23,231; 24,056; 24,290; 24,673 and 25,101) were found, corresponding to the amino acids 25, 167, 216, 514, 528, 557, 832, 910, 1037, 1180, respectively. In the literature, *Rocheleau *et al. has described an intra-individual variability early in 2021, mainly on spike domain, with a positive correlation between high variability per nucleotide location and gene length^29^. They detected, among 15,289 Sars-CoV-2 genomes analyzed, high frequency intra-host variability on codon 194, 215, 261, 655, 1254, 1258 and 1259 in spike domain, that represent a close region to our super variant codons and seems to be in similar distribution, close to key mutations E484, N501 per example. *Agius *et al. identified kinds of high variables clouds near to the mean VOC mutations, considering a potential role of those variability strand in deep mutational process, linked with strong interactions with our immune system^27^. In their interesting works, intra-host variability was the most important in ORF1a domain and in spike domain as we found for spike and NSP3 domain.

In our cohort, initial population were different on age and severity, which could have an important impact on conclusions, instead of no link was found between age and variability in our linear regression analysis. Patients suffering from malignancies, immunosuppressive treatment face higher COVID-19 related mortality risk and longer viral shedding. Although *Laubscher *et al. showed no more quasi-species rising in 6 patients from oncological department ^31^, our high throughput analysis showed higher number of subvariants in persistent shedding, and those discrepancies could be explained with the fewer number of patients than in our study. Moreover, they did not include samples collected after 3 weeks from diagnosis.

Diabetes mellitus constituted a large part (30%, n = 22) of our persistent patients compared to the non-persistent, and we did not conduct any subgroup analysis toward this part. To our knowledge, studies working on quasi-species in diabetic patients with acute COVID-19 has not been reported yet in literature, and still be built to understand deeper the intra-host SARS-CoV-2 evolution. We also saw differences between persistent and non-persistent intra-host genomic variability in mild patient, which confers reliability because persistent viral shedding has been related in mild patients to interact longer with host immune system^32^. *Al Khatib *et al. have interestingly found a such higher intra-host variability in severe patient, which differs with our results, likely because there were not severe patient enough in control group so we cannot conclude with significant difference^26^.

Furthermore, our study suffers from biases, residing in the fact that the ARTIC protocol is a source of significant variability. The use of the Oxford Nanopore technology is indeed characterized by a higher per-base error rate than short-reads sequencing techniques. Unless we circumvented this using a dedicated bioinformatic pipeline to avoid amplification errors (unpublished source), the genome’s depth we obtained is such that these errors are, at the end, in similar quantities to other NGS techniques. In fact, the majority of viral quasi-species studies use Illumina technology, which is described as more reliable^11^, and we demonstrate here the feasibility of in-depth analysis with Nanopore technology.

Important finding in this work may consist of N501Y and P681H mutation presence in spike domain, in high percentage on samples from 20A clade, sampled before Alpha (20I) or Omicron (21 K) variants rising. Although not all minority variants may emerge as VOC, intensive sequencing and analysis of SARS-CoV2 quasi-species by NGS, especially in persistent patients, would allow to anticipate potential future variants spreading^8^. As a matter of fact, SARS-CoV-2 cellular entry, which is effective thanks to spike protein and ACE2 receptor, can be dramatically changed by a single different nucleotide, the latter changing the entire 3D conformation of the target to its receptor^33^. Moreover, not only can cell biologists now predict the conformational structure of a nucleotide in the spike domain as a result of mutations, but also the viral target-cell receptor affinity resulting from those modifications^34^, which remains extremely sensitive as studies revealed a particular links between Sars-Cov-2 celerity of cellular entry and clinical severity^35^. We strongly encourage teams to involve quasi-species analysis on variant of concern massive surveillance, as we could keep one step ahead fill our quiver with another arrow.

## Conclusion

We found significant differences in global number of quasi-species clouds between persistent and non-persistent patient, which validates the hypothesis of persistent viral shedding patient could be a variant nursery. Further studies are absolutely needed to characterize variant virus “farmers” and provide clues for variant hunters.

## Materials and methods

### Collection samples

Among the thousand daily SARS-CoV-2 samples taken routine screening centralized at the IHU Mediterranean infection, APHM, Marseille, France, we prospectively and randomly selected 205 nasopharyngeal samples positive in SARS-CoV-2 real-time polymerase chain reaction. Samples selection was conducted from a routine sample list levied from March 2020 to August 2021. Inclusion conditions were designed as follow: to be older than 18 years, to have an RT-PCR positive test for SARS-CoV-2, regardless of clade, with Cycle threshold (Ct) between 10 and 34, regardless of comorbidities or treatment, regardless of duration of symptoms and stage of disease severity. Only patients with two positive PCR tests at least 17 days apart were selected, and up to 90 days to avoid including samples from re-infection. Randomization was done informatically from a list of patients who meet all inclusion criteria. For control population, we have selected positive SARS-CoV-2 nasopharyngeal samples as the same way, with randomization from a list which belong to the routine sequencing in our center. Inclusion criteria was viral clearance up to 17 days.

### Sequencing protocol

Samples that were positive for SARS-CoV-2, identified by real-time PCR with a Ct-

value from 10 to 34, were processed for next-generation sequencing. Whole genome sequencing was performed following the Eco PCR tiling of SARS-CoV-2 virus with native barcoding (Oxford Nanopore, version PTCE_9122_v109_revB_10feb2020). 200 μL of nasopharyngeal swab fluid after viral RNA extraction with the EZ1 Virus Mini Kit v2.0. Briefly, cDNA was synthesized from 10 μL of viral RNA using the LunaScript RT SuperMix kit (NEB, USA) with random hexamers. PCR was performed using Q5 Hot Start High-Fidelity DNA Polymerase (NEB, USA) and a set of primers targeting regions of the SARS-CoV-2 genome designed by the ARTIC network (https://artic.network/ncov-2019). The PCR mixture was initially incubated for 30 s at 98 °C for denaturation, followed by 35 cycles of 98 °C for 15 s and 65 °C for 5 min. The purified DNA was repaired with NEBNext Ultra II End Repair (NEB, USA), followed by DNA end preparation using NEBNext Ultra II End repair/dA-tailing Module (NEB, USA) and the successive attachment of native barcodes and sequencing adapters supplied in the EXP- NBD196 kit (Oxford Nanopore Technologies, UK) to the DNA ends. The DNA concentration was determined with a Qubit 3.0 instrument using a dsDNA HS Assay Kit (Thermo Fisher, USA). Repaired and “endpreped” products were pooled (480 µL for 48 samples) and purified with 192 µL of AMPure XP beads (Beckman Coulter, USA) and Short Fragment Buffer (NEB, USA) to exclude small nonspecific fragments. After priming the flow cell, 20 ng of DNA per sample of the products was pooled in a DNA library with a final volume of 12 μL. GridION Mk1 was used to perform genome sequencing in an virgin R9.4.1 flow cell from 2 to 4 h (depending on run quality and reads obtained).

### Bioinformatic analysis

Base calling was performed by using guppy (https://community.nanoporetech.com). High Accuracy Model (flip-flop) with the parameter settings “-c dna_r9.4.1_450bps_hac. cfg -x auto”, different samples were separated, and adapters were trimmed with the additional parameter settings “-trim_barcodes -barcodes EXP-NBD104/EXP-NBD114/EXP-NBD196”. FASTQ reads were filtered for quality control according to a cutoff “length ≥ 200 and Phred value ≥ 7” using the program “filtlong v0.2.0” (https://github.com/rrwick/Filtlong). Reads between 400 and 700 base pairs were kept; thus, potential chimeric reads were removed using artic pipeline (https://artic.network/ncov-2019). Selected reads were mapped against SARS-CoV-2 reference (Genbank accession no: NC_045512) using Minimap2 (v2.9). Sam2 consensus were used to sort the aligned BAM files, to obtain coverage data and a consensus sequence. Consensus sequences were extracted with a minimum depth coverage at 150X and stringency 70%. After we share the mapping (BAM files) on CLC Genomics workbench v.7 software. Data were inspected and alignment statistics were also calculated with CLC Genomics workbench v.7 software. All sequencings obtained were deposed on GISAID website (https://www.gisaid.org/) or in Genbank on the submission number: SUB11504102 (https://www.ncbi.nlm.nih.gov/genbank/).

#### Nucleotide variation representation (supp data)

SARS-CoV-2 genomes and the reference genome (NC_045512.2) were aligned using MAFFT v.7 (Katoh and Standley, 2013) before using snipit tool (https://github.com/aineniamh/snipit) that summarises SNPs relative to the given reference genome.

#### Phylogenetic analysis with whole genome

Phylogenetic trees were constructed using the nextstrain/ncov tool (https://github.com/nextstrain/ncov) and visualized with Auspice (2.36.0) software (https://auspice.us/). Pangolin lineage was added from a tsv file in the Auspice interface.

### Quasi-species analysis

Genomic variability was assessed for each sample using an in-house Excel matrix available on supplementary data (Supplementary Table 1). Sequencing format used were on “.TSV” from CLC Genomic workbench v.7, then copy and paste on the in-house matrix which can define, for every position, the proportion of variant reads from every nucleotide, in percentage value (for each position: % of A, T, C, G and deletion). We define stable variant quasi-species if variability on a specific position was higher than 25%, as previously describe^14^. The threshold for position of interest at 25% was also chosen following a tangent line on repartition of variability for all samples (Supplementary Fig. 1). Intra-host variability was thus defined by difference of 25% in nucleotides repartition given by genomic position.

We assessed and found hot spots of variations defined by more than 50% samples with a genomic variation > 24% for one given position (supplementary Fig. 1).

### Ethical statement

Whole genome sequencing was performed on nasopharyngeal samples that were collected in the context of routine diagnosis and not for research purpose. No additional samples were actively collected for this study. Clinical data were retrospectively retrieved from medical files and anonymized before analysis, only in the Assistance Publique-Hôpitaux de Marseille site and all methods were carried out in accordance with respecting the French GPDR (General Data Protection Regulation). Experimental protocol has been approved by the IRB research department unit from Assistance publique-Hôpitaux de Marseille under the number PADS-BJP737. No human genome has been sequenced. In line with the European General Data Protection Regulation No 2016/679, patients were informed of the potential use of their medical data and that they could refuse the use of their data. No ethical approval requirement was needed other than informed consent.

### Statistical analyses

Statistical analyses were carried out using Prism 9 for macOs (Version 9.1.1 (223), April 16, 2021, GraphPad Software, LLC, URL: https://www.graphpad.com). Categorical variables are presented as numbers and percentages, and continuous variables are presented as the means ± SD (standard deviation). Comparative analyses of the means of variabilities between persistent and non-persistent patients were built with Graphpad Software multiple comparison tools, using nonparametric Welch’s t-test or ANOVA. Comparative analyses between percentages were conducted with Chi-square or Fisher’s exact tests when appropriate. Alpha risk was considered for a *p value* > 0.05.

## Supplementary Information

Supplementary Information 1.

Supplementary Information 2.

Supplementary Information 3.

Supplementary Information 4.

Supplementary Information 5.

Supplementary Information 6.

Supplementary Information 7.

Supplementary Information 8.

Supplementary Information 9.

Supplementary Information 10.

Supplementary Information 11.

Supplementary Information 12.

## Data Availability

All data are available on demands following the correspondant author mail address. Supplementary figures and tables are given in the present article. The datasets generated and analysed during the current study are available in the PRJEB55073 repository, and in the following link : https://www.ebi.ac.uk/ .
