# Supplementary figures and images for: SARS-CoV-2 quasi-species analysis from patients with persistent nasopharyngeal shedding
Source: Sci Rep. 2022 Nov 4;12:18721. doi: 10.1038/s41598-022-22060-z (PMC9636146; doi:10.1038/s41598-022-22060-z)

# Threshold method

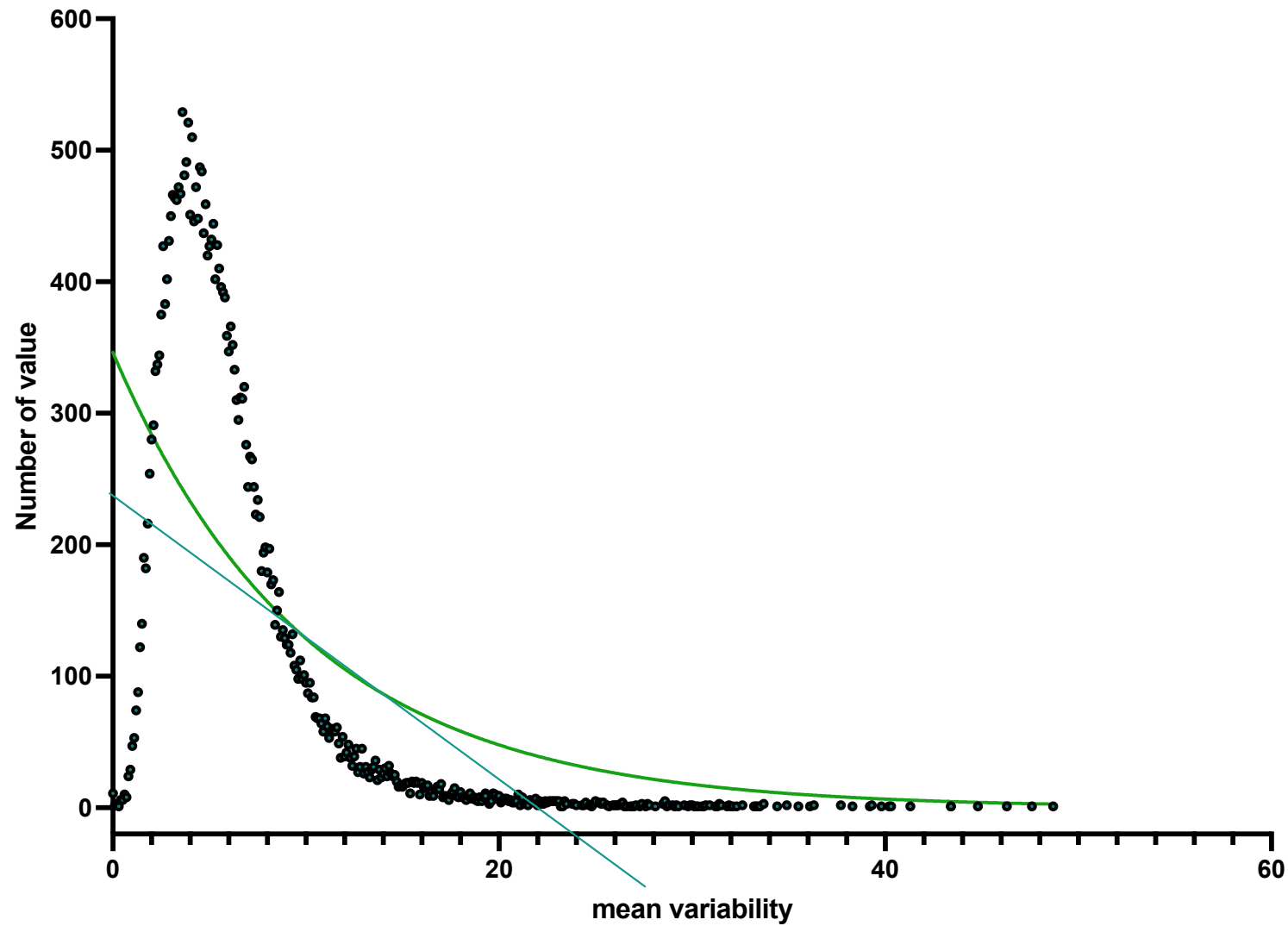

Supplement: Supplementary file 1 — Supplementary Information 1. [file 41598_2022_22060_MOESM1_ESM.pdf]

Correlation between Ct and mean of the reads per position/sample

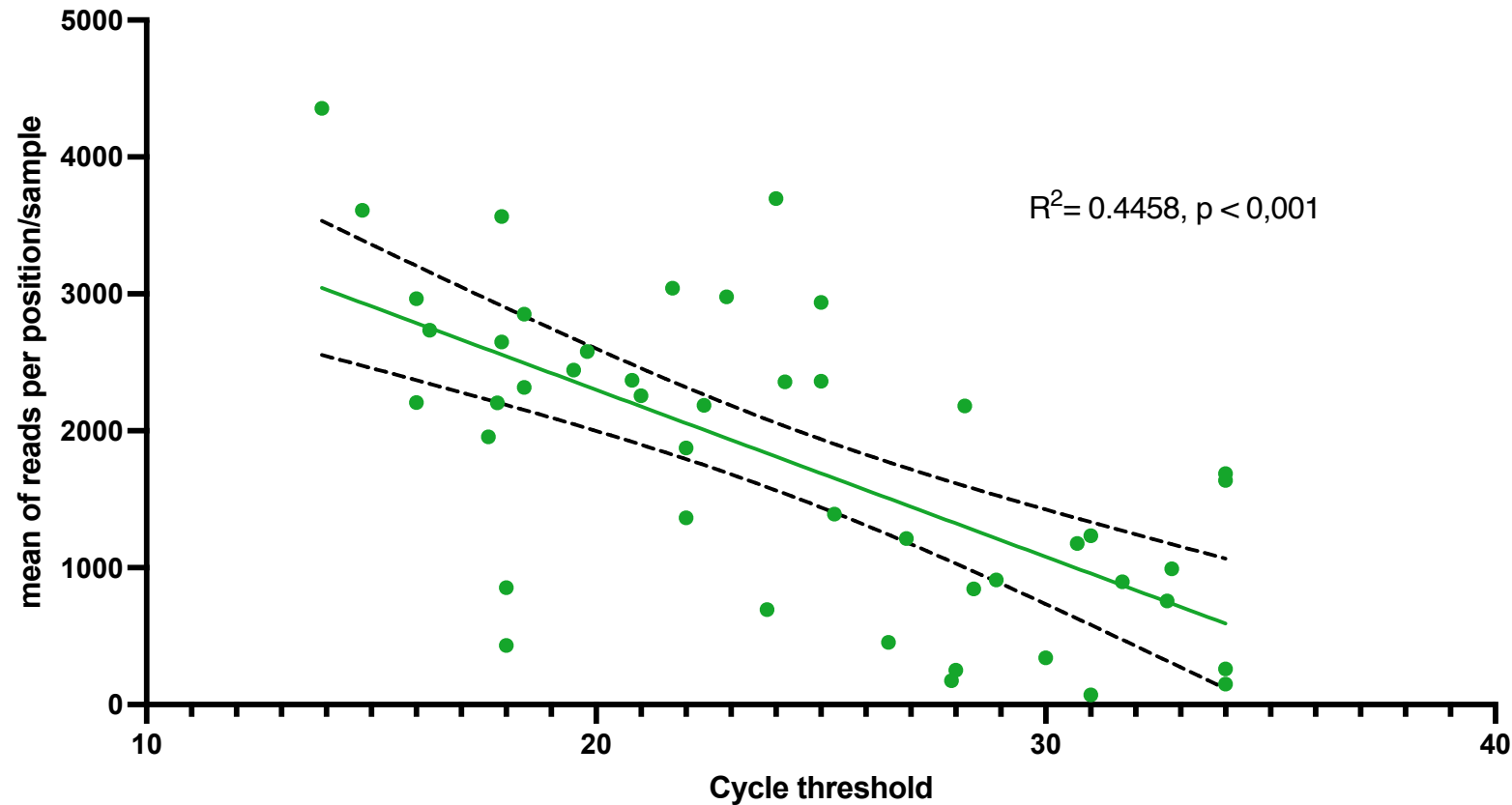

Supplement: Supplementary file 3 — Supplementary Information 3. [file 41598_2022_22060_MOESM3_ESM.pdf]

Correlation between variability per position and Ct

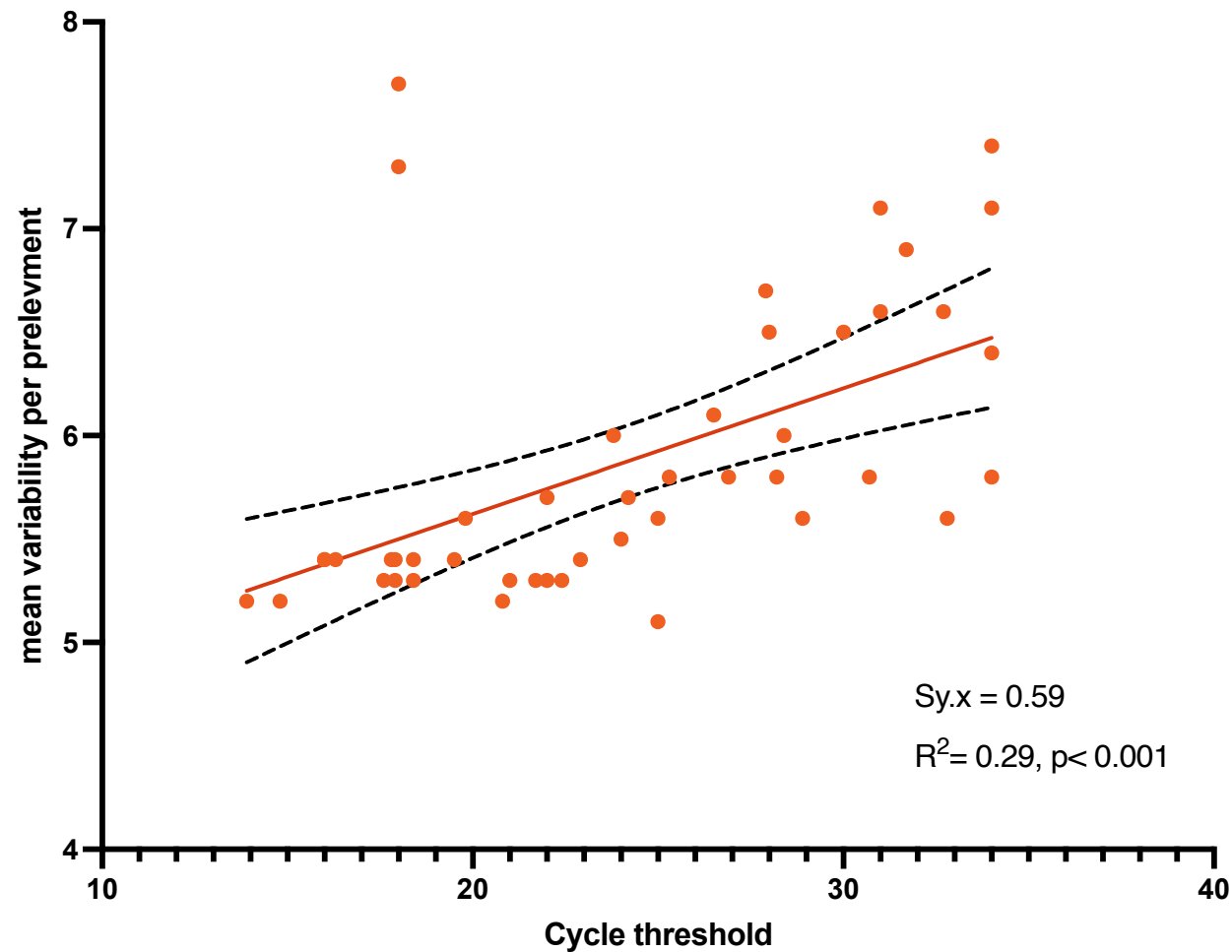

Supplement: Supplementary file 4 — Supplementary Information 4. [file 41598_2022_22060_MOESM4_ESM.pdf]

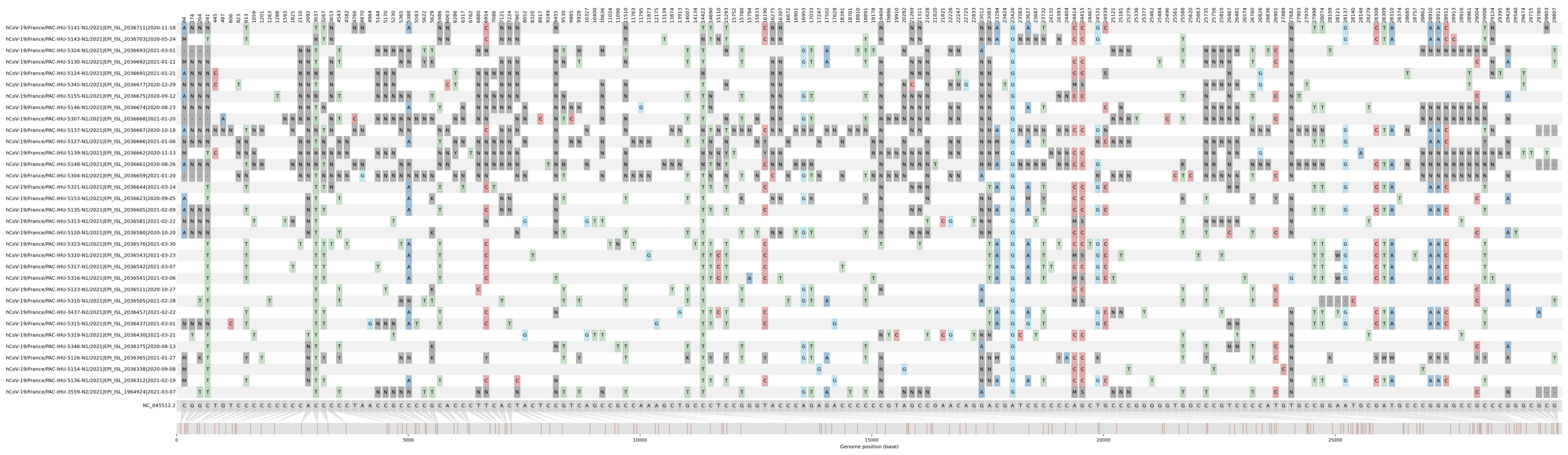

Supplement: Supplementary file 5 — Supplementary Information 5. [file 41598_2022_22060_MOESM5_ESM.png]

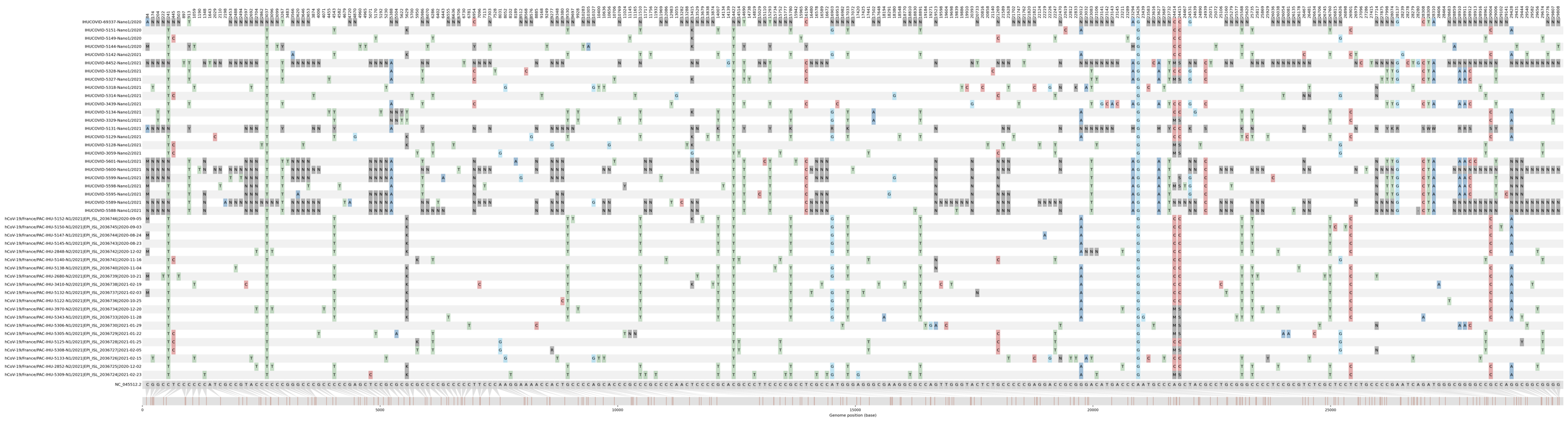

Supplement: Supplementary file 6 — Supplementary Information 6. [file 41598_2022_22060_MOESM6_ESM.png]
