# Supplementary material for: SARS-CoV-2 quasi-species analysis from patients with persistent nasopharyngeal shedding
Source: Sci Rep. 2022 Nov 4;12:18721. doi: 10.1038/s41598-022-22060-z (PMC9636146; doi:10.1038/s41598-022-22060-z)
Supplement: Supplementary file 8 — Supplementary Information 8. [file 41598_2022_22060_MOESM8_ESM.docx]

|  |  | |  |  | |  |  | | |  | |  | |  |
| --- | --- | --- | --- | --- | --- | --- | --- | --- | --- | --- | --- | --- | --- | --- |
| Sample | | Total reads (nb) | | SARS-CoV-2 reads (nb) | coverage (%) | Deletion (nb) | | | Mutation (nb) | | additional mutations | | Clade | |
| 1 | | 164293 | | 161785 | 99,89 | 0 | | 24 | | | A10652G,A24389C,A9274G,C13275T,C20844T,C21575T,C23086T,C24865T,C2862T,C3646T,C6636T,G22104T,G24390C,G9277T | | 20E (EU1) | |
| 2 | | 190354 | | 170711 | 99,06 | 0 | | 17 | | | A24389C,C12073T,C15738T,G14500T,G17427T,G21255C,G24390C | | 20E (EU1) | |
| 3 | | 187949 | | 181923 | 99,89 | 0 | | 22 | | | A24389C,C18877T,G22199A,G24390C,G25563T | | 20A.EU2 | |
| 4 | | 35377 | | 5040 |  |  | |  | | |  | | NRE | |
| 5 | | 35599 | | 18921 |  |  | |  | | |  | | NRE | |
| 6 | | 132109 | | 119610 | 92,90 | 4 | | 29 | | | A24389C,C84A,G24390C | | 20I/501Y.V1 | |
| 7 | | 313870 | | 302882 | 99,89 | 0 | | 23 | | | A24389C,C18877T,G24390C,G25563T,G28655T,T9376C | | 20A.EU2 | |
| 8 | | 188079 | | 184028 | 99,89 | 0 | | 18 | | | A24389C,C15951T,C23266T,G24390C,G28436T | | 20E (EU1) | |
| 9 | | 154145 | | 149040 | 99,52 | 0 | | 26 | | | A24389C,C18877T,C23188T,C26013T,C2704T,C29627T,C3096T,G24390C,G25563T | | 20A.EU2 | |
| 10 | | 261459 | | 259253 | 99,89 | 0 | | 29 | | | A24389C,A4870G,A8576G,C13824T,C18877T,C21682T,C25904T,G18534T,G24390C,G25563T,G25595C,T2029C | | 20A.EU2 | |
| 11 | | 124964 | | 122578 | 99,30 | 2 | | 28 | | | A24389C,A2692T,A8052G,C10632T,C21614T,C22792T,C9344T,G10396T,G24390C,T23560C | | 20H/501Y.V2 | |
| 12 | | 195898 | | 192824 | 99,89 | 5 | | 34 | | | A20379G,A24389C,C12970T,C21727T,G24390C,T16939C | | 20I/501Y.V1 | |
| 13 | | 50798 | | 32301 | 73,49 | 0 | | 19 | | | A24389C,C1288T,C18877T,C84A,G24390C,G25563T,G27877T | | 20A.EU2 | |
| 14 | | 135061 | | 64836 | 65,20 | 2 | | 14 | | | C23604A,C23709T,C27972T,C5388A,C5986T,G13993T,G24914C,G28048T | | 20B | |
| 15 | | 91212 | | 60522 | 81,31 | 0 | | 20 | | | A24389C,C18877T,G24390C,G25459T,G25563T | | 20A.EU2 | |
| 16 | | 145172 | | 142153 | 99,90 | 0 | | 24 | | | A24389C,C18877T,C26873T,C29073T,G24390C,G25563T,T26790C | | 20A.EU2 | |
| 17 | | 73016 | | 63969 | 89,97 | 0 | | 24 | | | A24389C,C16887T,C18877T,C84A,G15919T,G24390C,G25563T,G29511T,T26442C | | 20A.EU2 | |
| 18 | | 173980 | | 164559 | 99,90 | 2 | | 21 | | | A24389C,C25047T,G24390C | | autre | |
| 19 | | 266814 | | 264545 | 99,90 | 1 | | 17 | | | A24389C,A8031G,C15738T,G14500T,G17427T,G24390C | | 20E (EU1) | |
| 20 | | 90190 | | 81494 | 95,06 | 4 | | 32 | | | A24389C,G24390C,T7867C | | 20I/501Y.V1 | |
| 21 | | 266485 | | 210255 | 98,42 | 0 | | 24 | | | A24389C,C13860T,C18877T,C5170T,G24390C,G25563T,T6880C | | 20A.EU2 | |
| 22 | | 40306 | | 12781 |  |  | |  | | |  | | NRE | |
| 23 | | 95026 | | 81963 | 93,43 | 0 | | 17 | | | A24389C,C5388A,C84A,G24390C,G28346T | | 20A.EU2 | |
| 24 | | 107025 | | 94739 | 96,18 | 1 | | 19 | | | A24389C,C1201T,C18877T,C24381A,G24390C,G25563T | | 20A.EU2 | |
| 25 | | 41597 | | 32336 |  |  | |  | | |  | | NRE | |
| 26 | | 133955 | | 132185 | 99,89 | 0 | | 27 | | | A24389C,C13515T,C18877T,C222T,C25792T,C27688T,C503T,G24390C,G25563T,G26720T | | 20A.EU2 | |
| 27 | | 96453 | | 88612 | 78,74 | 4 | | 23 | | | A24389C,C84A,G24390C | | 20I/501Y.V1 | |
| 28 | | 74743 | | 53907 | 72,38 | 2 | | 12 | | | A11782G,C23604A,C23709T,C27972T,C28253T,C5388A,C5986T,G24914C,G28048T | | 20A | |
| 29 | | 155782 | | 152925 | 99,89 | 1 | | 30 | | | A24389C,C1059T,C13860T,C16575T,C17634T,C18877T,G18756T,G19684T,G24390C,G25563T,G28378A,G29553T,T19584C,T25081C,T2597C,T7096C | | autre | |
| 30 | | 316742 | | 314363 | 99,89 | 1 | | 26 | | | A24389C,C18877T,C23188T,C26013T,C2704T,C29627T,C3096T,G24390C,G25563T | | 20A.EU2 | |
| 31 | | 159138 | | 147049 | 99,08 | 0 | | 24 | | | A24389C,C15720T,C18877T,C2488T,G24390C,G25563T,G26153T | | 20A.EU2 | |
| 32 | | 225147 | | 167134 | 99,18 | 0 | | 24 | | | A24389C,C5388A,C84A,G24390C | | 20A.EU2 | |
| 33 | | 237032 | | 235274 | 99,08 | 0 | | 29 | | | A24389C,A24614G,C18877T,C4455T,C5192T,G24390C,G25563T | | 20A.EU2 | |
| 34 | | 200732 | | 196900 | 99,89 | 1 | | 21 | | | A24389C,C18877T,G24390C,G25563T | | 20A.EU2 | |
| 35 | | 201461 | | 198699 | 99,89 | 0 | | 28 | | | A24389C,A28213G,C18877T,C26954T,G11743T,G24390C,G25563T,G29511T,G29747T,G3566A,T26442C | | 20A.EU2 | |
| 36 | | 146767 | | 25058 | 59,14 | 0 | | 12 | | | A24389C,C15738T,C29686T,C29774T,C6762T,G24390C,G28122A | | 20E (EU1) | |
| 37 | | 268342 | | 265363 | 99,90 | 0 | | 23 | | | A24389C,C13665T,C18877T,C9693T,G24390C,G25563T | | 20A.EU2 | |
| 38 | | 169703 | | 151529 | 99,89 | 0 | | 22 | | | A24389C,C18877T,C26873T,C29073T,G24390C,G25563T,T26790C | | 20A.EU2 | |
| 39 | | 60420 | | 31897 | 58,67 | 2 | | 17 | | | A24389C,A28111G,A28281T,C14676T,C15279T,C23271A,C3267T,C84A,C913T,C9246T,G21800T,G24390C,G28280C,T16176C,T24506G,T28282A | | 20A | |
| 40 | | 117631 | | 95987 | 95,89 | 0 | | 12 | | | A17233G,A20268G,A24389C,C25254T,C25731T,C28833T,G24390C,G27463C | | autre | |
| 41 | |  | |  |  |  | |  | | |  | |  | |
| 42 | | 99216 | | 73551 | 86,06 | 1 | | 7 | | | A24389C,C5388A,C84A,G24390C | | 20A | |
| 43 | | 107329 | | 51375 | 80,20 | 0 | | 12 | | | A24389C,C28657T,C29366T,C84A,G24390C | | 20E (EU1) | |
| 44 | | 40150 | | 11502 |  |  | |  | | |  | | NRE | |
| 45 | | 117983 | | 100267 | 86,14 | 4 | | 28 | | | A24389C,C84A,G24390C | | 20I/501Y.V1 | |
| 46 | | 78807 | | 61738 | 65,18 | 4 | | 20 | | | A24389C,A28111G,A28281T,C14676T,C15279T,C23271A,C28977T,C3267T,C84A,C913T,G24390C,G28280C,T16176C,T24506G,T28282A,T6954C | | 20B | |
| 47 | | 155731 | | 120665 | 83,75 | 3 | | 24 | | | A24389C,A28111G,A28281T,C14676T,C15279T,C18877T,C23271A,C28977T,C3267T,C913T,G13126T,G24390C,G25563T,G28280C,G28884C,T16176C,T24506G,T28282A,T6954C | | 20B | |
| 48 | | 134279 | | 18553 |  |  | |  | | |  | | 20I/501Y.V1 | |
| 49 | | 145218 | | 144685 | 96,50485437 | 0 | | 20 | | | C18877T,C26735T,G11083T,G25563T, | | Marseille4 | |
| 50 | | 5236 | | 2480 | 7,753598929 | 0 | | 1 | | | NRE | | NRE | |
| 51 | | 15621 | | 225 | 100 | 0 | |  | | | NRE | | NRE | |
| 52 | | 10261 | | 534 | 1,359223301 | 0 | | 0 | | | NRE | | NRE | |
| 53 | | 8019 | | 3351 | 7,03046535 | 0 | | 2 | | | NRE | | NRE | |
| 54 | | 202960 | | 201516 | 99,88952126 | 0 | | 25 | | | A23416G,C18877T,C26735T,C29415T,C6555T,G18180A,G25552T,G25563T,G28280A, | | Marseille4 | |
| 55 | | 351630 | | 350728 | 99,89621694 | 0 | | 27 | | | A24383G,A24389C,C18877T,C26735T,G18462T,G24390C,G25563T,G28655T,G29759T,T24380A,T9376C, | | Marseille4 | |
| 56 | | 5043 | | 403 | 100 | 0 | |  | | | NRE | | NRE | |
| 57 | | 62270 | | 45132 | 74,97823904 | 0 | | 14 | | | A22705G,A6063C,C27944T,C823T,G23401T, | | Marseille2 | |
| 58 | | 314617 | | 313988 | 99,8928691 | 0 | | 27 | | | C18877T,C23188T,C25844T,C26013T,C26735T,C2704T,C29627T,C3096T,C4071T,G25563T, | | Marseille4 | |
| 59 | | 17376 | | 7017 | 18,60729829 | 0 | | 1 | | | NRE | | NRE | |
| 60 | | 299795 | | 299249 | 99,89 | 1 | | 17 | | | A8031G,C15738T,G14500T,G17427T,G5950T,T13402G, | | Marseille2 | |
| 61 | | 16160 | | 1128 | 3,89 | 0 | | 2 | | | NRE | | NRE | |
| 62 | | 73877 | | 39711 | 57,30 | 0 | | 17 | | | A16261C,A4870G,C18877T,C26735T,G25563T,G25595C,T25536C, | | Marseille4 | |
| 63 | | 260721 | | 259707 | 99,11 | 0 | | 18 | | | C11020T,C26029A,G26056A,G5504A, | | Marseille2 | |
| 64 | | 12730 | | 833 | 3,00 | 0 | | NA | | | NRE | | NRE | |
| 65 | | 257581 | | 256919 | 99,20 | 0 | | 18 | | |  | | 20B | |
| 66 | | 16516 | | 12697 | 35,58 | 0 | | 7 | | | C18877T,G25563T, | | Marseille4 | |
| 67 | | 52472 | | 38155 | 65,57 | 0 | | 19 | | | C18877T,C26735T,G25311T,G25471C,G25563T,G487A,T4766C,T8914C,T9861C, | | Marseille4 | |
| 68 | | 36794 | | 10433 | 29,73 | 0 | | 10 | | | C18877T,C26735T,C27389T,G25563T, | | Marseille4 | |
| 69 | | 233741 | | 232859 | 99,14 | 1 | | 16 | | | A8031G,C15738T,G14500T,G17427T,G5950T, | | Marseille2 | |
| 70 | | 12696 | | 5862 | 18,49 | 0 | | 5 | | | NRE | | NRE | |
| 71 | | 350335 | | 349735 | 99,08 | 0 | | 26 | | | C10765T,C18877T,C26735T,G25563T, | | Marseille4 | |
| 72 | | 4510 | | 1402 | 2,55 | 0 | | 0 | | | NRE | | NRE | |
| 73 | | 218141 | | 215411 | 100,00 | 0 | | 31 | | | A17406G,C11704T,C11919T,C1348T,C14805T,C15763T,C16658T,C18796T,C18877T,C23086T,C26735T,G16564T,G25563T,G8102T,T5071C, | | Marseille4 | |
| 74 | | 299147 | | 297668 | 98,30264479 | 2 | | 28 | | | A7121T,C1263T,C16658T,C18877T,C26735T,G25563T, | | Marseille4 | |
| 75 | | 262418 | | 260606 | 98,69099431 | 0 | | 31 | | | C12049T,C13207G,C13806T,C15960T,C17999T,C18877T,C21219T,C23647T,C26735T,G25563T,G27877T,G4913A,G5194A,G6513A,T733C, | | Marseille4 | |
| 76 | | 421817 | | 420380 | 99,8928691 | 0 | | 32 | | | C12049T,C13207G,C13806T,C15960T,C17999T,C18877T,C21219T,C23647T,C26735T,G25563T,G27877T,G4913A,G5194A,G6513A,G9053T,T733C, | | Marseille4 | |
| 77 | | 228822 | | 228378 | 97,55942417 | 4 | | 34 | | | A28095T,G25352T,G25785T,G29751A,T13899G,T15096C, | | Anglais | |
| 78 | | 91413 | | 88937 | 90,04017409 | 2 | | 18 | | | A8052G,C10632T,C1593T,C21614T,G10396T, | | SudAfricain | |
| 79 | | 183679 | | 183386 | 98,39973217 | 1 | | 18 | | | A13042G,A18366G,C11152T,C26029T,C3874T,C5700T,C9165T, | | 20E (EU1) | |
| 80 | | 248451 | | 247908 | 97,27485772 | 4 | | 33 | | | C5583T,T606C, | | Anglais | |
| 81 | | 208923 | | 208005 | 99,88617342 | 4 | | 37 | | | C16293T,C19164T,C25096T,C26549T,C29095T,C9451T,G15780A,T15096C,T27656G, | | Anglais | |
| 82 | | 339831 | | 338472 | 99,89621694 | 4 | | 35 | | | A24389C,C1825T,C18377T,C24109T,C5144T,G24390C,T15096C, | | Anglais | |
| 83 | | 292037 | | 291317 | 99,08269166 | 0 | | 27 | | | C18687T,C18877T,C26735T,G25563T, | | Marseille4 | |
| 84 | | 242789 | | 240172 | 98,52025444 | 3 | | 26 | | | A2692T,A8052G,C10632T,C21614T,G10396T,G19872T,T19896C,T20802C,T23560C, | | SudAfricain | |
| 85 | | 123640 | | 122986 | 96,96350854 | 2 | | 26 | | | A24389C,A2692T,A8052G,C10632T,C21614T,G10396T,G19872T,G24390C,T19896C,T23560C, | | SudAfricain | |
| 86 | | 309030 | | 308392 | 99,8928691 | 4 | | 35 | | | A11860G,C25156T,C25638T,C28728T,C8320T,G25785T,T15096C, | | Anglais | |
| 87 | | 271166 | | 232618 | 94,45262805 | 5 | | 32 | | | A24389C,C6317T,C7086T,G24390C, | | Anglais | |
| 88 | | 213522 | | 212995 | 98,41981922 | 0 | | 30 | | | A1992G,A2161G,C106T,C18877T,C19032T,C2062T,C26735T,G10870T,G21941T,G22346T,G25563T,G26062T,G29734T,T27410C, | | Marseille4 | |
| 89 | | 202726 | | 201308 | 99,11282223 | 3 | | 41 | | | A24389C,C11020T,C11750T,C14120T,C19390T,C2110T,C21297T,C24370T,C24442T,C3653T,C4582T,C5365T,G24390C, | | Anglais | |
| 90 | | 111502 | | 110390 | 81,13491798 | 0 | | 23 | | | A26811T,C18796T,C18877T,C19164T,C26735T,G25563T, | | Marseille4 | |
| 91 | | 66998 | | 59829 | 79,08938735 | 2 | | 18 | | | A16534G,A26667G,C21191T,C6198T,G10396T,T23560C, | | SudAfricain | |
| 92 | | 172165 | | 171680 | 97,87412119 | 2 | | 27 | | | A16534G,A26667G,A2692T,A8052G,C10632T,C21191T,C21614T,C6198T,G10396T,T23560C, | | SudAfricain | |
| 93 | | 305673 | | 305012 | 99,8928691 | 0 | | 33 | | | C10702T,C14724T,C23086T,C27849T,C4455T, | | Anglais | |
| 94 | | 377593 | | 376060 | 99,8928691 | 4 | | 33 | | | A24389C,A8568C,C7528T,G24390C,T21126C, | | Anglais | |
| 95 | | 26355 | | 9084 | 30,02678273 | 4 | | 8 | | | NRE | | NRE | |

NRE : no read enough
