# Supplementary material for: SARS-CoV-2 quasi-species analysis from patients with persistent nasopharyngeal shedding
Source: Sci Rep. 2022 Nov 4;12:18721. doi: 10.1038/s41598-022-22060-z (PMC9636146; doi:10.1038/s41598-022-22060-z)
Supplement: Supplementary file 12 — Supplementary Information 12. [file 41598_2022_22060_MOESM12_ESM.docx]

Supplementary table 1: analytic Matrix for quasi-species analysis.

Supplementary table 2: details on sequencing, persistent samples.

Supplementary table 3: details on GISAID or Genbank submission with accession numbers.

Supplementary Figure 1: threshold method.

Supplementary Figure 2: details on clades repartition according to Nextstrain analysis.

Supplementary Figure 3: a/linear regression between Ct value and number of reads per position. b/linear regression between Ct and mean variability per sample Simple linear regression test.

Supplementary Figure 4: details on mutations profile in persistent group with GISAID submission numbers.
